# Comprehensive evaluation of using mineral and bio phosphorus fertilization on orange tree (*Citrus sinensis*) productivity

**DOI:** 10.1016/j.heliyon.2024.e39603

**Published:** 2024-10-21

**Authors:** Ahmed M.E. Elazazi, El-Sayed A.M. Awad, Salah M. Dahdoh, Azza S. Hussein, Essam M.A. Radwan, Esraa M.E. Hussein, Hussein H.M. Saeed, Hesham S. Ghazzawy, Mohamed F.M. Abdelkader, Mohamed H. Mahmoud, Mostafa M. Gouda, Xiaoli Li, Mohamed A. Abdein, EL-Sayed M. Qaoud

**Affiliations:** a Soil Science Department, Faculty of Agriculture, Zagazig University, Zagazig, 44511, Egypt; b Pomology Department, Faculty of Agriculture, Assiut University, Assiut, 71526, Egypt; c Horticulture Department, Faculty of Agriculture, New Valley University, El-Kharga, Egypt; d Horticulture Department, Faculty of Agriculture, Sohag University, 82524, Egypt; e Horticultural Department, Faculty of Agriculture and Natural Resources, Aswan University, Egypt; f Central Laboratory for Date palm Research and Development, Agriculture Research Center, Giza, 12511, Egypt; g Department of Plant Production, College of Food and Agriculture, King Saud University, Riyadh, 12372, Saudi Arabia; h Department of Biochemistry, College of Science, King Saud University, Riyadh, 12372, Saudi Arabia; i College of Biosystems Engineering and Food Science, Zhejiang University, Hangzhou, 310058, China; j Department of Nutrition and Food Science, National Research Centre, Dokki, Giza, 12422, Egypt; k Seeds Development Department, El-Nada Misr Scientific Research and Development Projects, Turrell, Mansoura, 35511, Egypt; l Horticultural Department, Faculty of Agriculture, Suez Canal University, 41522, Egypt

**Keywords:** Orange productivity and quality, Mineral and bio phosphorus fertilization in orange trees

## Abstract

Phosphorus (P) is considered as an essential element in crop production. Besides, the study of P from the elemental and bio sources impacts on productivity and quality of orange trees could emphasize its utilization importance. This study aimed to evaluate the chemical implication of three treatments of P fertilization (Triple phosphate (TP; 620 and 900 g/tree/year), phosphate rock (PR; 1820 and 2742 g/tree/year) and 1:1 TP:PR on Valencia orange trees (*Citrus sinensis* L. Osbeckunder). Where, all these treatments were equipped with bio-phosphorus fertilizer (phosphorin) (0 and 5 g/tree/year added twice in the year, Feb. and Jun.). The measurements included leaves nitrogen (N), phosphorus (P) and Potassium (K), number of fruits (NoF), juice weight/volume, total soluble solids (T.S.S.), vitamin C (V.C), and acidity. The results showed that the treatment (50 % TP +50 % PR at 450 + 1371g/tree/year + phosphourin at 5g/tree/year) gave the highest values for all characters i.e. N % in tree leaves 2.61 and 2.69 %, P% in tree leaves 0.16 and 0.17 %, K in tree leaves 1.79 and 1.86 %, number of fruits/tree 412.0 and 420.0, fruit weight 267.0 and 280.0g, fruit weight/tree 110.0 and 117.60 kg, fruit size 260.41 and 272.37 cm^3^, juice weight 114.1 and 126.15g, fruit juice 44.33 and 44.99 %, juice volume 110.15 and 114.90 cm^3^, peel thickness 0.58 and 0.60 cm, T.S.S. 12.14 and 12.21 %, T.S.S./Acid ratio 9.07 and 9.41, and V.C 70.15 and 72.44 (mg/100 ml) as well as the lowest total acidity 0.93 and 0.91 %, under both the 1st season and the 2^nd^ one, respectively. In conclusions, as the application of bio-fertilization has improved the quality charcatistics over seasons. Thus, this study opens the way towards the advanced application of bio-P element to understand the biogeochemical cycles, dynamics and function of natural ecosystems elements in agricultural and food security program.

## Graphical abstract

**Figure:**
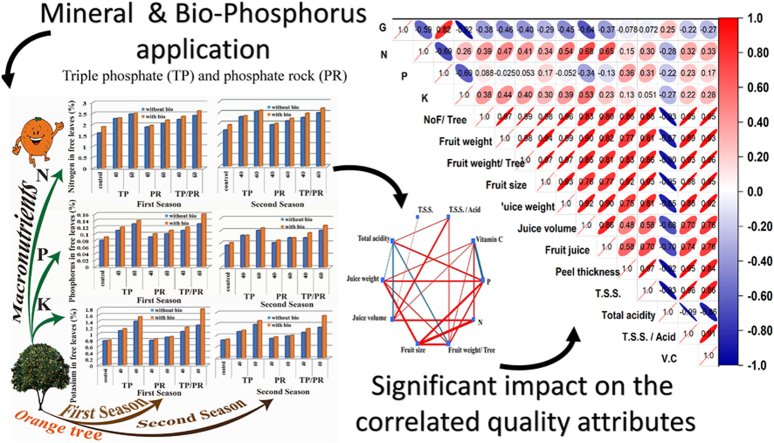

## 1 Introduction

Orange (*Citrus sinensis* L. Osbeck) have high economic impacts and are rated one of the most important fruit crops for exportation in Egypt [1]. Where it is among the richest sources of vit. C [2,3]. Most of the newly reclaimed soils are cultivated with fruit crops, particularly citrus. Newly reclaimed soils are poor in their nutrients, organic matter, and lower in keeping water, with high nutrient losses by leaching, resulting in lower nutrient uptake by plants and then causes a negative influence on growth, efflorescence, fruit yield and quality. So, Orange trees planted in that soils require excessive awareness in cultural treatments such as fertilization to improve the growth, yield and fruit quality [4]. For example, the mineral phosphorus (P) fertilizers possess the potential to enhance agricultural productivity significantly and play a crucial role in sustaining the burgeoning global populace. Nevertheless, intricate edaphic mechanisms lead to the immobilization of P within the soil matrix, thereby obstructing its prompt and adequate accessibility for absorption by vegetative organisms. The resulting limited utilization efficiency of contemporary water-soluble P fertilizers engenders considerable ecological and public health challenges. Furthermore, the extant methodologies aimed at augmenting P use efficiency have proven insufficient in mitigating these issues [5].

As P is an important nutrient needed by plants, its great function in releasing of energy through cellular metabolism [6,7]. The influence of phosphorus on fruit quality not be clear yet, although a reduce in soluble solids content and total acidity were correlated with phosphorus application which revealed that phosphorus fertilization had a significant effect on orange yield quality [8]. This agronomic application could have a technological influence on the consumer acceptance of the produced orange. For instance, its concentration of vit. C that has antimicrobial influence could affect its postharvest storage ability, where the orange, like most fresh products, is susceptible to postharvest losses, the most significant of which are juice %, water content, planting, and cultivation growth factors [9]. The fertilization with bio phosphorus had a major role of citrus production as it increases yield and fruit quality [10,11]. The bio-fertilization treatments with phosphourin were significantly surpassing the other fertilizer applications, particularly the control for the number of leaves, and their contents of nitrogen, phosphorous and potassium [12]. Adekiya, Dahunsi [13] reported that biofertilizers are integrated with the minerals’ significant effects on leaf contents of N, P, and K, yield components, number of fruits/tree comparing with both bio or minerals only. Therefore, the aim of this study was to evaluate the effect of different phosphorus sources and levels as well as bio fertilization on the produced orange fruit quality, and yield as well as opens the way towards the advanced application of P fertilization for enhancing the orange productivity and quality. We elucidate multiple methods that substantiate the bio-P fertilizer formulations and their corresponding application methodologies for enhancing P uptake and its food industrial related applications.

## 2 Materials and methods

### 2.1 Experimental site and agricultural practices

Two experiments were achieved over 2020/2021 and 2021/2022 seasons on 13 – year-old Valencia orange (*Citrus sinensis* L. Osbeck) trees budded on Folka Marina orange rootstock to study the effect of mineral and bio phosphorus fertilization on yield and quality characters of summer orange. The trees were cultivated in a citrus field at Salheya town (30.746557°N 32.00449°E), Sharkia Governorate, Egypt. The planting distances were 5 × 4 m. The irrigation system was drip irrigation. the practices recommended to the commercial citrus fields were followed.

### 2.2 Treatments

Mineral phosphorus fertilization:

Three treatments of phosphorus fertilization were used as followed.

1 Triple phosphate (TP) 37 % P_2_O_5_ with two levels (40 and 60 units represented 620 and 900 g/tree/year added in four times).
2 phosphate rock (PR) 12.5 % P_2_O_5_ with two levels (40 and 60 unit represented 1820 and 2742 g/tree/year added once.
3 50 % TP +50 % PR with two levels (40 and 60 unit represented 910 + 310 and 450 + 1371g/tree/year).

Triple phosphate and phosphate rock were presented from Abu Zaabal Fertilizers And Chemical Company.*Bio phosphorus fertilization*.

Bio phosphorus fertilizer (phosphorin), (P-dissolved bacteria) produced by General Organization for Agriculture Equalization Found (GOAEF), Ministry of Agriculture, Egypt, was applied in two levels (0 and 5 g/tree/year added twice at February and June). The quantities of mineral and bio phosphorus fertilizers are shown in (Table 1).

**Table 1:** Phosphorus fertilization treatments.

Phosphorus treatments	Phosphorus (P) rate (g/tree/year)	
	40 unit	60 unit
Triple Phosphate (TP)	620	900
Phosphate Rock (PR)	1820	2742
50 % TP +50 % PR	310 + 910	450 + 1371
Bio phosphorus (Phosphorin)	Added twice at February and June in a rate of 5 g/tree	

### 2.3 Measurements

#### 2.3.1 Fruit yield

At harvesting date of summer orange fruits, the fruits on each tree were picked to estimate: number of fruits/tree and total yield/tree (kg/tree). The physical and the chemical characteristic of the soil are presented in Table (2).

**Table 2:** The physical and the chemical characteristic of the soil.

Depth (cm)	Particle size (%)			Textural Class	Ph	EC dsm^−1^	N content mg kg^−1^	
	Sand	Silt	Clay				NO_3_-	NH_4_+
0–20	70.7	22.55	6.80	∗∗Ls	8.41	0.54	38	87
20–40	93.8	4.34	1.82	∗S	8.45	0.37	64	48
40–60	96.4	2.81	0.76	∗S	8.33	0.29	59	47

*Depth (cm)*	*cations mmolcL*^*−*^*^1^*				*Anions mmolcL*^*−*^*^1^*			
	Ca++	Mg++	Na+	K+	CO_3_-	HCO_3_-	SO_4_-	Cl-
0–20	1.66	0.91	2.02	0.81	0	2.4	1.17	1.83

20–40	1.02	0.58	1.67	0.43	0	1.78	0.58	1.34
40–60	0.83	0.49	1.24	0.34	0	1.13	0.64	1.13

#### 2.3.2 Nitrogen, phosphorus and potassium contents in leaves

The measurement of N, P, K were according to modified methods of Bilbao, Giraldo [14] and Pan, Zhang [15]. In brief, the tree leaves were collected in September 2020 and 2021, and then dried at 70 °C. After that, 0.2 g of the ground leaves was digested in a mix of concentrated sulfuric and perchloric acids (4: 1 v/v) for 20 min, then transferred quantitatively to 50 ml. Nitrogen in orange leaves was measured by using macro Kjeldahl and Phosphorus in orange leaves was measured by spectrophotometer as well as Potassium in orange leaves was measured by using flame-photometer.

#### 2.3.3 Fruit characteristics

The measurement of fruit characteristics were following Singh, Chahal [16]. Ten fruits ripened were randomly taken from every replicate to estimate peel thickness (cm), fruit weight (g) and size (cm^3^), then juice volume/fruit (cm^3^) was estimated.

#### 2.3.4 Total acidity

The collected fruit juice total acidity was estimated as citric acid equivalent due to calibration versus 0.1 NAOH solution and then calculated following equation [1]:

[1] $$\text{Total}\;\text{acidity}=\left[\frac{\text{NAOH}\times 0.1\times 0.064}{\text{Juice}\;\text{volume}\;\left({cm}^{3}\right)}\right]\times 100$$

#### 2.3.5 Total soluble solids percentage (T.S.S.%)

Total soluble solids percentage (T.S.S.%): was estimated by the refractometer following Zhang, Zhan [9].

T.S.S./total acidity ratio was calculated following equation [2]:

[2] $$T.S.S.\;/\text{TA}\;\%=\left(\frac{T.S.S.\%}{\text{Total}\;\text{acidity}\%}\right)\times 100$$

#### 2.3.6 Vitamin C content (V.C)

Vitamin C content was measured according to Al-Qurashi and Awad [17] and Ranganna [18]. In brief, the samples were measured by spectrophotometric method and the concentrations were calculated as mg ascorbic acid/100 ml juice from the calibration curve versus 2, 6-dichlorophenol endophenol dye.

### 2.4 Statistical analysis

the experimental design was factorial in completely randomized block design with 3 replicates, the data were analyzed by using MINITAB software package (Minitab Inc., State College, USA). The data were analyzed and presented as means ± standard deviation (SD). One-way ANOVA was used to calculate the means' differences signification using Duncan's multiple range test at *P* < 0.05 and 95 % confidence limit. Also, multivariate analysis and principle components analysis (PCA) were calculated by using R-package and Matlab R2017b (MathWorks, USA) [19,20].

## 3 Results

**Fig. 1:**
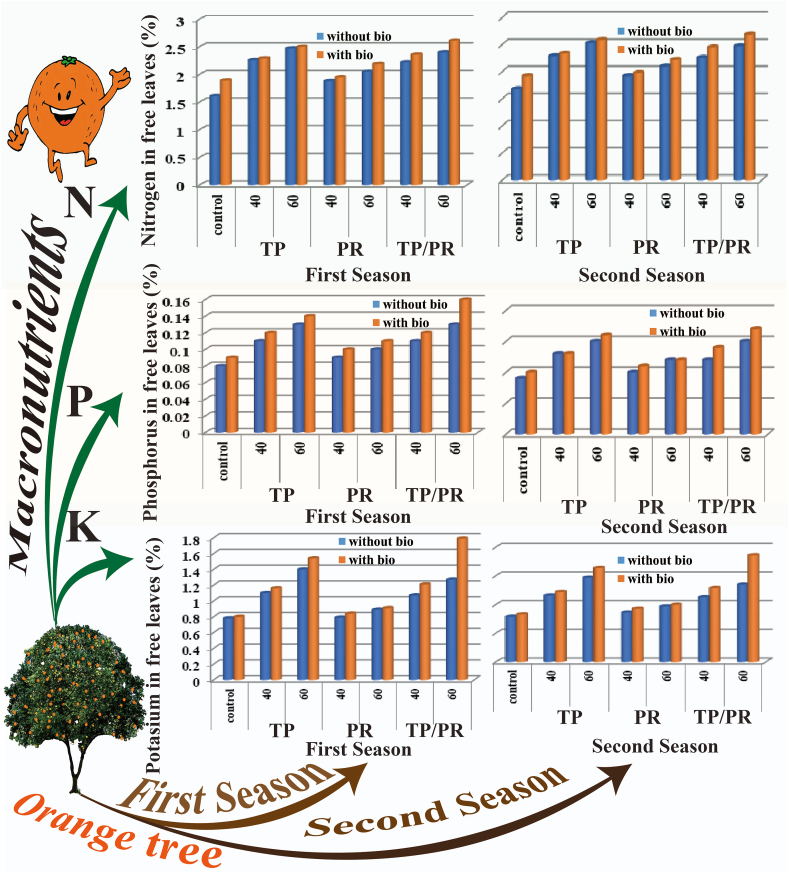
Phosphorus and bio fertilization impact on the orange trees' macronutrients.

***Mineral Phosphorus fertilization effects*** As N, P, K are vital nutrients required for optimum productivity growth of plants, where normally the macro-nutrients like N, P, and/or K not only show the interacting pathways for each other but also affect the other micro-nutrient pathways [21]. In this study, the macronutrients results revealed that tree leaves NPK% were affected significantly from the different P treatments (Fig. 1). The highest estimates of N and K% in tree leaves (2.51 and 2.58 %) and (1.53 and 1.61 %) were recorded by the treatment of (50 % Triple Phosphate+50 % Phosphate rock) (50 % TP+50 % PR) at a level of 60 unit/tree/year, whereas, the highest values of P% in tree leaves (0.17 and 0.16 %) were registered by Triple Phosphate at a level of 60 unit/tree/year (TP at 60 unit), under both the 1st season and 2^nd^ one, respectively (Table 3).

**Table 3:** Effect of phosphorus and bio fertilization on N, P and K% in tree leaves under 2020 and 2021 seasons.

Source	P %	N% in tree leaves						P% in tree leaves						K% in tree leaves					
		1st season			2nd season			1st season			2nd season			1st season			2nd season		
		Without bio	With bio	Mean	Without bio	With bio	Mean	Without bio	With bio	Mean	Without bio	With bio	Mean	Without bio	With bio	Mean	Without bio	With bio	Mean
Control		1.61^i^	1.89^h^	1.75^e^	1.68^f^	1.92^e^	1.80^e^	0.08^e^	0.10^de^	0.09^c^	0.09^f^	0.11^ef^	0.10^c^	0.78^f^	0.80^ef^	0.79^cd^	0.79^h^	0.83^h^	0.81^d^
TP	40	2.26^cde^	2.29^cde^	2.28^b^	2.29^c^	2.34^b^	2.32^b^	0.11^bcd^e	0.13^bcd^	0.13^b^	0.13^bcde^	0.13^bcde^	0.10^b^	1.10^de^	1.16^cd^	1.13^b^	1.16^f^	1.22^ef^	1.19^b^
	60	2.47^ab^	2.50^ab^	2.49^a^	2.53^b^	2.59^ab^	2.56^a^	0.13^abc^	0.15^ab^	0.17^a^	0.15^abc^	0.17^ab^	0.16^a^	1.40^bc^	1.54^b^	1.47^a^	1.47^c^	1.64^b^	1.56^a^
PR	40	1.88^h^	1.95^gh^	1.92^d^	1.92^e^	1.98^de^	1.95^d^	0.09^de^	0.11^cde^	0.10^bc^	0.10^ef^	0.12^def^	0.11^bc^	0.79^f^	0.84^f^	0.82^d^	0.86^h^	0.93	0.90^d^
	60	2.05^fg^	2.19^ef^	2.12^c^	2.10^de^	2.22^cd^	2.16^c^	0.10^cde^	0.12b^cde^	0.11^bc^	0.12^cdef^	0.12^cdef^	0.12^bc^	0.89^f^	0.91^f^	0.90^c^	0.97^h^	1.00^gh^	0.99^c^
TP + PR	40	2.22^de^	2.36bcd	2.29^b^	2.26^c^	2.45^b^	2.36^b^	0.11^bcde^	0.13^bcd^	0.12^b^	0.12^cdef^	0.14^abcd^	0.13^b^	1.07^de^	1.21^cd^	1.14^b^	1.13 ^fg^	1.29^de^	1.21^b^
	60	2.40^bc^	2.61^a^	2.51^a^	2.47^b^	2.69^a^	2.58^a^	0.13^abc^	0.17^a^	0.15^a^	0.15^abc^	0.17^a^	0.16^a^	1.27^c^	1.79^a^	1.53^a^	1.35^d^	1.86^a^	1.61^a^
Mean		2.13^a^	2.26^a^	2.19	2.18^a^	2.31^a^	2.25	0.11^b^	0.13^a^	0.12	0.12^b^	0.14^a^	0.13	1.0^4b^	1.18^a^	1.11	1.10^b^	1.25^a^	1.18
L.S.D 0.05		P: 0.11, Bio: 0.08			P: 0.11, Bio: 0.07			P: 0.02, Bio: 0.01			P: 0.02, Bio: 0.01			P: 0.21, Bio: 0.06			P: 0.10, Bio: 0.05		
		P × Bio 0.15			P × Bio 0.14			P × Bio 0.03			P × Bio 0.03			P × Bio 0.14			P × Bio 0.11		

Mean with different alphabet superscript within the same column and analytical parameter indicate that values differ significantly.

The initial difference in the tree production characteristics were associated with the change in the NPK%. An increase in all production was observed when the combination of TP and PR was used compared to each group of treatments. For instance, as shown in Table 4 that the variation in the number of fruits/tree (NoF) was changed from 331.0 to 420.0 in the second season after using the biofertilization compared to the mineral P control with 201. Where, fruit weight and fruits weight/tree were changed from 176 g to 34.141 kg/tree to 257.50 g and 97.91 kg/tree after using 60 % of TP:PR (50 % ratio).

**Table 4:** Effect of phosphorus and bio fertilization on number of fruits/tree, fruit weight and fruit weight/tree under 2020 and 2021 seasons.

P Source	P Rate	Number of fruits/tree						Fruit weight (g)						Fruit weight/tree (kg)					
		1st season			2nd season			1st season			2nd season			1st season			2nd season		
		No bio	With bio	Mean	No bio	With bio	Mean	No bio	With bio	Mean	No bio	With bio	Mean	No bio	With bio	Mean	Without bio	With bio	Mean
Control		194^jk^	200^j^	197.0^d^	201^k^	210^jk^	205.5^d^	176^h^	179^h^	177.50^d^	178.0^jk^	180^jk^	179.50^e^	34.14^l^	35.80^l^	34.97^d^	35.78^k^	37.80^k^	36.79^e^
TP	40	281^f^	301^e^	291.00^b^	289^g^	309^f^	299.00^b^	214^f^	221^e^	217.50^b^	220^g^	228^f^	224.00^b^	60.13^g^	66.52^f^	63.33^b^	63.58^g^	70.45^f^	67.02^b^
	60	363^b^	394^a^	378.50^a^	372^c^	404^b^	388.00^a^	240^c^	260^b^	250.00^a^	258^c^	271^b^	264.50^a^	87.12^c^	102.44^b^	94.782^a^	95.98^c^	109.48^b^	102.73^a^
PR	40	192^k^	205^j^	198.50^e^	202^k^	214^j^	208.00^d^	177^h^	183^h^	180.00^d^	182^k^	190i^j^	186.00^d^	33.98^l^	37.52^k^	35.75^d^	36.76^k^	40.66^f^	38.71^d^
	60	222i	241^h^	231.50^c^	232	249i	240.50^c^	188	200^g^	194.00^c^	195i	207^h^	201.00^c^	41.74^j^	48.20i	44.97^c^	45.24^j^	51.54i	48.39^c^
TP + PR	40	263^g^	324^d^	293.50^b^	272^h^	331^e^	301.50^b^	209^f^	233^d^	221.00^b^	216^g^	240^e^	228.00^b^	54.97^h^	75.49^e^	65.23^b^	58.24^h^	79.44^e^	68.84^b^
	60	346^c^	412^a^	379.00^a^	355^d^	420^a^	387.50^a^	248^d^	267^a^	257.50^a^	251^d^	280^a^	265.50^a^	85.81^d^	110.0^a^	97.91^a^	89.11^d^	117.60^a^	103.36^a^
Mean		265.9^b^	296.7^a^	281.93	274.71^b^	305.29^a^	290.00	207.4^b^	220.4^a^	213.9	214.3^b^	228.0^a^	221.2	56.84^b^	68. 0^a^	62.42	60.67^b^	72.42^a^	66.55
L.S.D0.05		P:10.1,Bio:2.5			P: 8.9, Bio:2.5			P: 5.2, Bio:3.3			P: 6.0, Bio:2.8			P: 2.8, Bio:2.7			P: 2.7, Bio:2.1		
		P × Bio 10.4			P × Bio 9.3			P × Bio 6.3			P × Bio 6.7			P × Bio 4.7			P × Bio 3.6		

Mean with different alphabet superscript within the same column and analytical parameter indicate that values differ significantly.

The different effect of the non or biofertilization had a significant influence on the juice %, weight, and volume (Table 5). Where, the juice % was changed in the first season from 36.441 % for the control to 44.33 % after using 60 % of TP:PR (50 % ratio). Besides, the peel thickness of 40 % P that used TP:PR (50 % ratio) was significantly increased to 0.56 cm compared to the control with 0.47 cm (Table 6). That was in association with, T.S.S. (11.95 and 12.03 %), fruit size (246.58 and 257.04 cm^3^) and V.C (68.04 and 68.97 (mg/100 ml)) as well as the best total acidity (0.95 and 0.94 %) that were recorded by the treatment of (50 % Triple Phosphate+50 % Phosphate rock) (50 % TP+50 % PR) at a level 60 unit/tree/year under both 1st season and 2^nd^ one, respectively (Table 7). Furthermore, the treatment of Triple Phosphate (TP at 60 units) gave the highest estimates of P% in tree leaves (0.17 and 0.16 %) (Table 3), The best fruit juice (43.46 and 44.02 %) and juice volume (10.77 and 107.58 cm^3^) (Table 5), as well as T.S.S./acid ratio (8.66 and 8.94) (Table 6) under both 1st season and 2^nd^ one, respectively. On the contrast, the worst estimates of the abovementioned characters were obtained by control belonging with Phosphate Rock (PR) at levels 40 unit.

**Table 5:** Effect of phosphorus and bio fertilization on Fruit juice, Juice weight (g) and juice volume under 2020 and 2021.

P Source	P Rate	Fruit juice (%)						Juice weight (g)						Juice volume (cm^3^)					
		1st season			2nd season			1st season			2nd season			1st season			2nd season		
		Without bio	With bio	Mean	Without bio	With bio	Mean	Without bio	With bio	Mean	Without bio	With bio	Mean	Without bio	With bio	Mean	Without bio	With bio	Mean
Control		36.44^l^	38.06^l^	37.25^f^	36.58^j^	38.74^h^i^j^	37.66^e^	67.20^kl^	68.85^jk^	68.03^d^	70.03^jk^	71.71^j^	70.87^e^	61.78^l^	65.45^j^	63.62^e^	63.14^jk^	66.32i	64.73^d^
TP	40	40.12^de^	40.73^cd^	40.43^b^	40.91^de^	41.54^cd^	41.23^bc^	85.73^g^	89.95^f^	87.84^b^	90.09^g^	94.91^f^	92.50^c^	82.61^g^	86.31^f^	84.46^b^	89.62^f^	89.88^f^	89.75^b^
	60	43.10^ab^	43.82^a^	43.46^a^	43.85^ab^	44.19^ac^	44.02^a^	105.58^c^	113.27^b^	109.43^a^	113.18^c^	119.81^b^	116.50^a^	99.69^c^	105.84^b^	102.77^a^	104.56^c^	110.60^b^	107.58^a^
PR	40	38.08^f^	38.40^f^	38.24^c^	38.96^e^	39.33^e^	39.15^d^	67.51^l^	70.40^jk^	68.96^d^	70.75^k^	74.56^l^	72.66^e^	62.52^l^	66.23^k^	64.38^d^	64.93^k^	69.55^j^	67.24^e^
	60	38.66^ef^	38.93^ef^	38.80^c^	39.72^e^	40.24^de^	39.98^cd^	72.86^j^	77.74i	75.30^c^	77.60i	83.32^h^	80.46^d^	70.21^j^	74.22i	72.22^c^	74.22i	76.94^h^	75.58^c^
TP + PR	40	39.55^ef^	41.48^bcd^	40.52^b^	40.57^de^	42.84^bc^	41.71^b^	82.81^h^	96.61^e^	89.71^b^	87.77^g^	102.78^e^	95.28^b^	78.14^h^	91.22^e^	84.68^b^	81.67^g^	94.05^e^	87.86^b^
	60	41.97^bc^	44.33^a^	43.15^a^	43.04^bc^	44.99^a^	44.02^a^	107.10^d^	114.0^a^	110.55^a^	108.16^d^	126.15^a^	117.16^a^	95.27^d^	110.15^a^	102.71^a^	99.63^d^	114.90^a^	107.27^a^
Mean		39.70^b^	40.82^a^	40.45	40.52^b^	41.70^a^	41.34	84.11^b^	90.12^a^	87.12	88.23^b^	96.18^a^	92.20	78.60^b^	85.63^a^	82.69	82.12^b^	88.89^a^	854.72
L.S.D0.05		P:1.23,Bio 0.97			P:1.53,Bio:0.86			P:2.10,Bio 1.74			P: 2.67,Bio:1.45			P: 2.43,Bio:1.30			P: 2.11,Bio:1.44		
		P × Bio 1.64			P × Bio 1.78			P × Bio 2.87			P × Bio 3.08			P × Bio 2.79			P × Bio 2.64		

Mean with different alphabet superscript within the same column and analytical parameter indicate that values differ significantly.

**Table 6:** Effect of phosphorus and bio fertilization on peel thick ness and T.S.S. (%) under 2020 and 2021 seasons.

P Source	P Rate	Peel thick ness (cm)						T.S.S. (%)						Total acidity (%)					
		1st season			2nd season			1st season			2nd season			1st season			2nd season		
		Without bio	With bio	Mean	Without bio	With bio	Mean	Without bio	With bio	Mean	Without bio	With bio	Mean	Without bio	With bio	Mean	Without bio	With bio	Mean
Control		0.46^m^	0.47^l^	0.47^f^	0.46^kl^	0.48^j^	0.47^f^	10.80^op^	10.92n	10.86^f^	10.86^op^	10.97^n^	10.92^e^	1.25^a^	1.23^cd^	1.24^a^	1.24^a^	1.18^bc^	1.21^a^
TP	40	0.52^de^	0.53^cd^	0.53^b^	0.53^de^	0.54^cd^	0.54^b^	11.46^e^	11.53^e^	11.50^c^	11.51^f^	11.58^f^	11.55^b^	1.00 ^fg^	0.99^g^	1.00^e^	1.03^e^	0.99^g^	1.01^d^
	60	0.56^b^	0.56^b^	0.56^a^	0.57^b^	0.58^b^	0.58^a^	11.84^b^	11.90^b^	11.87^b^	11.97^c^	12.08^b^	12.03^a^	0.96^f^	0.95^g^	0.96^f^	0.95^hi^	0.93^ij^	0.94^e^
PR	40	0.47^gh^	0.48^g^	0.48^d^	0.48^i^	0.49^hi^	0.49^d^	10.88^h^	11.04	10.96^e^	11.03^ij^	11.10^i^	11.07^d^	1.21^b^	1.15^c^	1.18^b^	1.15^bc^	1.12^cd^	1.14^b^
	60	0.48^g^	0.50^f^	0.49^c^	0.50^gh^	0.51 ^fg^	0.51^c^	11.15^g^	11.20^g^	11.18^d^	11.22^h^	11.27^h^	11.25^c^	1.13^cd^	1.09^d^	1.11^c^	1.09^de^	1.07^ef^	1.08^c^
TP + PR	40	0.51^ef^	0.54^c^	0.54^b^	0.52^ef^	0.55^c^	0.54^b^	11.31^f^	11.63^d^	11.47^c^	11.38^g^	11.70^e^	11.54^b^	1.05^e^	0.98^g^	1.02^d^	1.05^f^	0.97^gh^	1.01^d^
	60	0.54^c^	0.58^a^	0.56^a^	0.55^c^	0.60^a^	0.58^a^	11.75^c^	12.14^a^	11.95^a^	11.85^d^	12.21^a^	12.03^a^	0.97^gh^	0.93^h^	0.95^f^	0.97^gh^	0.91^j^	0.94^e^
Mean		0.51^b^	0.52^a^	0.52	0.52^b^	0.54^a^	0.53	11.31^b^	11.48^a^	11.40	11.40^b^	11.56^a^	11.48	1.08^a^	1.05^b^	1.06	1.07^a^	1.02^b^	1.05
L.S.D0.05		P: 0.004, Bio:0.001			P: 0.01, Bio:0.002			P: 0.06, Bio 0.04			P: 0.06, Bio:0.03			P: 0.04, Bio:0.01			P: 0.03, Bio:0.01		
		P x Bio 0.01			P x Bio 0.01			P x Bio 0.07			P x Bio 0.07			P x Bio 0.04			P x Bio 0.03		

Mean with different alphabet superscript within the same column and analytical parameter indicate that values differ significantly.

**Table 7:** Effect of phosphorus and bio fertilization on Fruit size, T.S.S./Acid ratio and V.C. under 2020 and 2021 seasons.

P Source	P Rate	Fruit size (cm3)						T.S.S./Acid ratio						Vit.C. (mg/100 ml)					
		1st season			2nd season			1st season			2nd season			1st season			2nd season		
		Without bio	With bio	Mean	Without bio	With bio	Mean	Without bio	With bio	Mean	Without bio	With bio	Mean	Without bio	With bio	Mean	Without bio	With bio	Mean
Control		154.46^mn^	162.48^j^	158.47^e^	161.90o	168.78^m^	165.34^e^	5.16^m^	6.12^l^	5.64^e^	5.35^m^	6.23^k^	5.79^e^	49.04^h^	51.22^gh^	50.13^d^	50.58^hi^	51.75^h^	51.17^d^
TP	40	193.74^f^	222.41^f^	208.08^b^	202.70^g^	231.37^f^	217.04^b^	7.21^f^	7.48^e^	7.35^b^	7.44^g^	7.76^f^	7.60^b^	58.55^d^	59.83^cd^	59.19^b^	60.08^de^	60.94^d^	60.51^b^
	60	239.41^c^	252.07^b^	245.74^a^	248.70^c^	261.70^b^	255.20^a^	8.42^b^	8.89^a^	8.66^a^	8.73^c^	9.15^b^	8.94^a^	65.93^b^	68.48^a^	67.27^a^	67.49^b^	70.39^a^	68.94^a^
PR	40	160.74^jk^	164.74	162.74^d^	168.70	175.04	171.87^d^	5.75^k^	6.00^j^	5.88^d^	5.89^k^	6.27^j^	6.08^d^	49.33^h^	51.20^gh^	50.27^d^	50.50^i^	52.60^gh^	51.55^d^
	60	172.74i	181.74^h^	177.24^c^	180.70^j^	189.70i	185.20^c^	6.30^i^	6.63^h^	6.47^c^	6.62^i^	6.83^i^	6.73^c^	52.95 ^fg^	55.04^ef^	54.00^c^	54.44 ^fg^	55.95^f^	55.20^c^
TP + PR	40	204.41^g^	215.07^e^	209.74^b^	213.040^h^	223.70^e^	218.37^b^	6.91^g^	7.84^d^	7.38^b^	7.18^h^	8.04^e^	7.61^b^	56.36^e^	61.65^c^	59.01^b^	58.05^e^	63.05^c^	60.55^b^
	60	232.74^d^	260.41^a^	246.58^a^	241.70^d^	272.37^a^	257.04^a^	8.11^c^	9.07^a^	8.59^a^	8.41^d^	9.41^a^	8.91^a^	65.93^b^	70.15^a^	68.04^a^	65.50^b^	72.44^a^	68.97^a^
Mean		194.03^b^	208.42^a^	201.23	204.7^b^	217.52^a^	210.01	6.84^b^	7.43^a^	7.14	7.09^b^	7.67^a^	7.38	56.60^b^	59.65^a^	58.13^b^	58.09^b^	61.02^a^	59.55
L.S.D0.05		P: 5.72, Bio:2.15			P: 5.53, Bio:2.59			P: 0.22, Bio 0.12			P: 0.19, Bio:0.13			P: 1.81, Bio:1.06			P: 1.78, Bio:0.97		
		P × Bio 6.12			P × Bio 6.15			P × Bio 0.26			P × Bio 0.24			P × Bio 2.14			P × Bio 2.06		

Mean with different alphabet superscript within the same column and analytical parameter indicate that values differ significantly.

### 3.1 Bio phosphorus fertilization effect

Regarding, bio Phosphorus fertilization, the results revealed that the application of bio fertilization improved the N, P, and K% in tree leaves by (6.10 and 5.96 %), (18.18 and 16.67 %) and (13.46 and 13.64 %) (Table 3); the number of fruits/tree, fruit weight and fruits weight/tree by (11.60 and 11.13 %), (6.27 and 6.40 %) and (19.63 and 19.37 %) (Table 4); the fruit juice, juice weight and juice volume by (2.82 and 2.91 %), (7.15 and 1.27 %) and (8.94 and 6.69 %) (Table 5); peel thickness and T.S.S. by (1.96 and 3.85 %) and (1.50 and 1.40 %) and decreased total acidity by (2.78 and 4.67 %) (Table 6) and the fruit size, T.S.S./Acid ratio and VC (mg/100 ml) by (7.42 %), (8.63 and 8.18 %) and (5.39 and 5.04 %) (Table 7) over without application under both 1st and 2^nd^ season, respectively.

### 3.2 Interaction effect

Regarding, P and bio fertilization interaction, both seasons showed close indications of the status of the different quality productions. In addition, the application of (50 % TP+50 % PR) at a level 60 unit with bio fertilization gave the best estimates of N% (2.61 and 2.69 %), P% (0.16 and 0.17 %) and K% (1.79 and 1.86 %) in tree leaves (Table 3); number of fruits/tree (412.0 and 420.0), fruit weight (267.0 and 280.0g) and fruits weight/tree (110.0 and 117.60 kg) (Table 4); fruit juice (44.33 and 44.99 %), juice weight (114.1 and 126.15 g) and juice volume (110.15 and 114.90 cm^3^). (Table 5); peel thickness (0.58 and 0.60 cm) and T.S.S. (%) (12.14 and 12.21 %) and total acidity (%) (0.93 and 0.91 %) (Table 6) and fruit size (260.41 and 272.37 cm^3^), T.S.S./acid acidity ratio (9.07 and 9.41) and VC (mg/100 ml) (70.15 and 72.44) (Table 7) in both the 1st season and 2^nd^ one, respectively. Meanwhile, the worst values were recorded by application of (PR at 40 unit) under without bio fertilization in both the 1st season and 2^nd^ one, respectively.

**Fig. 2:**
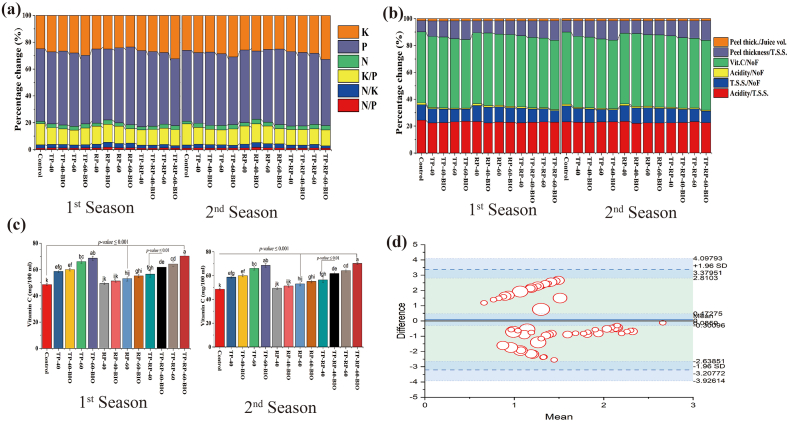
(a) Percentage change % of the Number of fruits/tree, Fruit weight (g), Fruit size (cm^3^), Fruit juice (%), Juice weight (g), and Juice volume (cm^3^) by different phosphorus fertilization. (b) Percentage change % of the Number of fruits/tree, Fruit weight (g), Fruit size (cm^3^), Fruit juice (%), Juice weight (g), and Juice volume (cm^3^) by different phosphorus fertilization. (c) Vitamin C implications from the different treatments. (d) Overall experimental variance of the entire studied groups.

**Fig. 3:**
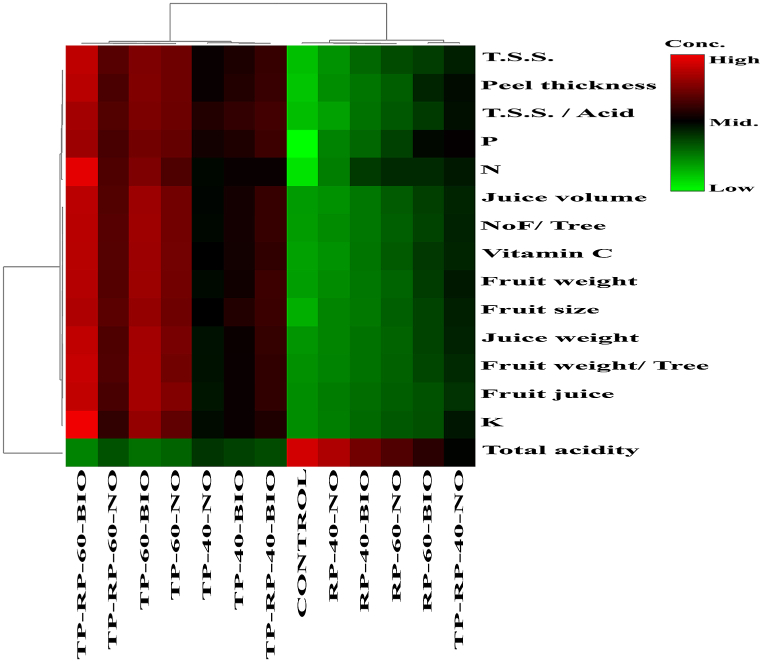
Heatmap of the experimental parameters and their clustered groups based on the treatments.

As shown in Fig. 2, the percentage changes (%) of the NPK and their ratios were changed according to the P type of bio and elemental fertilizations which mainly affected the K/P ratio with slight differences in the two under studied seasons (Fig. 2a). Besides, significant changes in the ratios of the quality production parameters (fruit weight, fruit size, fruit juice, peel thickness, juice weight, juice volume, TS.S., and vitamin C) were observed according to the changes in the P type fertilizations (Fig. 2b). Where, the Vitamin C: NoF/tree ratio was significantly increased when applied TP: RP 60 % with bio fertilization compared with the control of RP. Besides, for the Vitamin C, TP: RP resulted in an enhancement in V.C values throughout gradually increase the concentration from 40 % to 60 % (Fig. 2c). The increased rates of V.C was depending on the variations differences in the N% (Fig. 2d). It showed the superior behaviors in the biofertilization compared to the mineral P fertilization. Thus, the number of fruits/trees, fruit weight and fruits weight/tree, fruit juice, juice weight and juice volume were affected the Vit. C and total acidity that were affected by the application of different Phosphorus sources and levels under both seasons (Fig. 3).

### 3.3 Data multivariate analysis and overall correlation

**Fig. 4:**
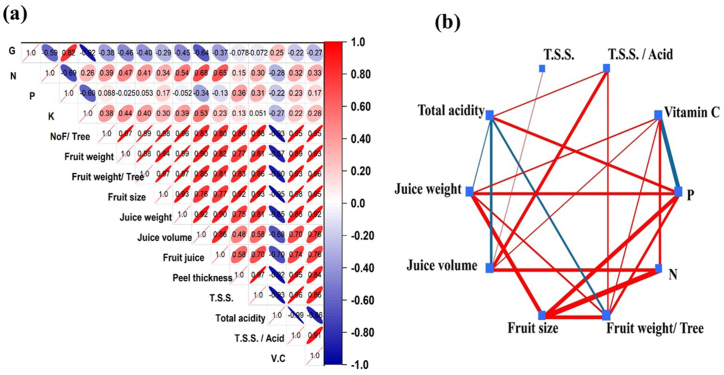
(a) Correlation heatmap among the entire features under study. (b) Map Network among the studied parameters principal components by PLSDA. The red color assigned for positive correlations and blue for negative correlations. Where, in the network the built correlations are considered (*P* < 0.05) and the thicker line means higher signification.

A heatmap was performed using Pearson's correlation in Fig. 4a. Through correlation analysis, the N% content had a significant positive correlation with juice volume (r^2^ = 0.68) and fruit juice % (r^2^ = 0.65), and negatively correlated with K (r^2^ = −0.69) and T.S.S. (r^2^ = −0.28). Also, P was positively correlated with peel thickness (r^2^ = 0.36). While it was negatively correlated with NoF/tree (r^2^ = −0.60) and total acidity (r^2^ = −0.28). Where, NoF/tree was positively correlated with peel thickness (r^2^ = 0.97) and negatively correlated with total acidity (r^2^ = −0.93). To better understand the contents linkages, a correlation network among the most important PLSDA loadings was performed (Fig. 4b). That Pearson network between the studied parameters at *P* < 0.05 signification level has proved the high positive signification impact of leaves N on the orange juice concentration of Vitamin C (*p*-value = 0.03). On the other hands, leaves' P% negative correlation (*p*-value = 0.007) with vitamin C was affected by the total acidity, juice weight, juice volume, and fruit size [19].

**Fig. 5:**
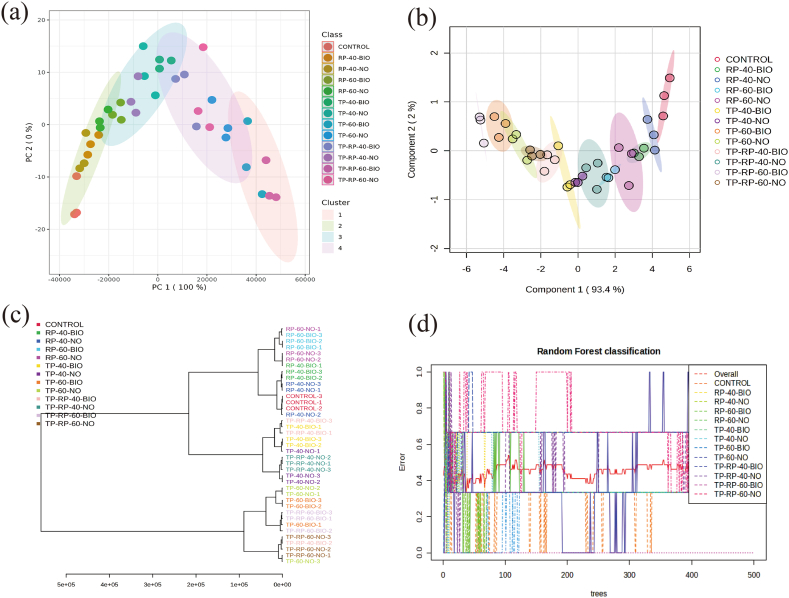
Multivariate analysis of the studied parameters by PLSDA. (a) k-mean cluster analysis among the overall groups. (b) Principle component Analysis (PCA) of the first group understudy. (c) similarity tree cluster analysis among the studied groups. (d) Random Forest (RF) cluster figure among the different treatments.

The k-mean clusters and principal component analysis (PCA) was prepared in Fig. 5 to find similarities and differences between the orange leaves NPK% changes and the quality parameters evaluation [20]. The k-mean clusters were extracted from 15 indexes, and the variance contributions of the total variance of the four groups (control, TP, RP, TP + RP) (Fig. 5a). Also, in Fig. 5b the variance contribution of components 1 and 2 was the highest (95.4 % in the TP and RP group). And it has showed a significant discrimination (*P* < 0.05) among the studied groups in both seasons based on the similarity tree discrimination (Fig. 5c). And it has decreased the PC1 and PC2 score when 60 % of TP and RP bio-fertilization was applied compared to the control group. Based on the random forest (RF) cluster that considered the error influences into the nonlinear clustering of the groups [22,23], the bio-P showed the highest influence through changing the inorganic elemental P to Bio-P quality parameters (Fig. 5d).

## 4 Discussion

Phosphorus is included in photosynthesis, respiration and the enzyme activity regulation [24]. This important element has a main function in the internal energy transport of plants, such phosphate esters. Majority of phosphate esters are interfering of biosynthesis and metabolic dissolution. As its formation and function are immediately relative to the tree plants energy metabolism [25]. Wu, Li [8] studied how phosphorus application influences on quality development, especially sucrose, fructose, glucose and citric acid contents. phosphorus application improved the fruit quality, through reducing titratable acid and increasing T.S.S., sugars and T.S.S./titratable acid ratio.

Several previous researchers discussed phosphorus fertilization in different sources and rates, and bio phosphorus fertilization in citrus trees severally [24,[26], [27], [28]]. But in the present study, the effect of phosphorus fertilization in different sources and rates, as well as bio phosphorus fertilization on citrus fruit yield and quality were evaluated simultaneously. The results revealed highly significant variations among the evaluated treatments in all studied measurements. Regarding phosphorus fertilization in different sources and rates, the treatment of (50 % Triple Phosphate+50 % Phosphate rock) (50 % TP+50 % PR) at a level 60 unit/tree/year obtained the best N%, and K% in tree leaves. Also, it enhanced the number of fruits/tree, fruits weight/tree, T.S.S./Acid ratio and Vit.C content the first and second seasons. Li, Li [10] reported that P fertilization with 49.5 kg hm^−2^ enhanced citrus yield by 32.6 % correlated with the enhance in fruit number. Whereas, the treatment of (TP at 50 unit) gave the highest P% in tree leaves in the 1st season and 2^nd^ one. The impact of phosphorus fertilization on fruit quality seem to be less evident, although a reduction in soluble solids and total acidity were correlated with phosphorus fertilization [5]. EL-Khwaga, Abd El-Latif [29] revealed that phosphorus fertilization had a significant impact on orange yield and quality. Importantly, the using of various forms of biofertilizers such, phosphorus solubilizers, and mobilizers, increasing citrus growth, through enhancing essential minerals resources, manufacture Siderophore, encourage phytohormone production [30]. In the present study, the application of bio fertilization improved the studied characteristics i.e., N, P, and K in tree leaves by (6.10 % and 5.96 %), (18.18 % and 16.67 %) and (13.46 % and 13.64 %).

That enhancements could be from the nutrients availability increase due to enhancing nitrogenase, chitinases, and glucanases activities by improve the microbial metabolism in soil which is important thing for citrus trees and increasing soil fertility [31]. Additionally, the bio-fertilization treatments with phosphourin was significantly surpassing the other fertilizer applications, particularly the control for the number of leaves, and their contents of nitrogen, phosphorous and potassium [32]. Mohamed and Massoud [33] reported that mineral biofertilizers had significant effects on leaf contents of N, P, and K, yield components, number of fruits/tree, fruit weight, juice volume, T.S.S.%, T.S.S./acid ratio, and reduced juice acidity % comparing with both bio or and mineral only.

With regard of the interaction between mineral P and its bio fertilization, the results showed that the application of (50 % TP+50 % PR) at a level 60 unit with bio fertilization gave the best values of all characters under the study compared with without-bio fertilization in both seasons. These results are in agreement with those obtained by Wu, Li [8,34] who reported that bio-P fertilization significantly enhances the citrus fruit's yield and quality.

## 5 Conclusions

The current study provides significant insights into the role of P fertilization (both mineral and bio-sources) in enhancing the productivity and quality of Valencia orange trees that suffer from P and N low accumulations from the soils. The application of bio-P could, besides increasing fruit tree performances, be a useful tool to improve soil fertility in intensive agriculture systems. Where, the study findings showed that the application of a combination of Triple P and phosphate rock, supplemented with bio-P fertilizer, leads to remarkable improvements in key growth parameters, including N, P, and K contents in leaves, as well as fruit yield and quality metrics such as weight and juice volume compared to typical inorganic P fertilizer for 3 years administration. This study not only highlights the efficacy of bio-P fertilization in enhancing orange tree productivity but also sets the stage for future research that could lead to more sustainable agricultural practices. This underscores the critical importance of P in agricultural practices aimed at sustaining crop productivity in nutrient-poor soils, particularly in newly reclaimed areas where nutrient leaching is prevalent. Future prospects should explore the long-term effects of various P fertilization strategies on soil health and crop sustainability. Additionally, developing more efficient multifaceted treatment systems that concern the ecological impacts of these fertilization methods will be crucial in promoting sustainable agriculture and ensuring food security.

## CRediT authorship contribution statement

**Ahmed M.E. Elazazi:** Investigation, Conceptualization. **El-Sayed A.M. Awad:** Investigation. **Salah M. Dahdoh:** Methodology. **Azza S. Hussein:** Methodology, Formal analysis. **Essam M.A. Radwan:** Investigation. **Esraa M.E. Hussein:** Investigation. **Hussein H.M. Saeed:** Methodology, Formal analysis. **Hesham S. Ghazzawy:** Investigation, Formal analysis, Conceptualization. **Mohamed F.M. Abdelkader:** Investigation, Formal analysis. **Mohamed H. Mahmoud:** Investigation. **Mostafa M. Gouda:** Writing – review & editing, Writing – original draft, Visualization, Validation, Supervision, Software, Resources, Project administration, Methodology, Investigation, Funding acquisition, Formal analysis, Data curation, Conceptualization. **Xiaoli Li:** Methodology, Formal analysis, Data curation. **Mohamed A. Abdein:** Investigation, Formal analysis, Data curation, Conceptualization. **EL-Sayed M. Qaoud:** Software, Project administration, Investigation, Funding acquisition, Formal analysis.

## Data and code availability

Data will be made available on request.

## Funding

This work was funded by the “Belt and Road” joint project fund between 10.13039/501100004835Zhejiang University, China, and 10.13039/100007787National Research Centre, Egypt (Project No: SQ2023YFE0103360). Also, it was supported by the 10.13039/501100002383King Saud University (Riyadh, Saudi Arabia) for the funding of this research through Researchers Supporting Project number (RSP-2024/R406).

## Declaration of competing interest

The authors declare that they have no known competing financial interests or personal relationships that could have appeared to influence the work reported in this paper.
